# The Ap-2α/Elk-1 axis regulates Sirpα-dependent tumor phagocytosis by tumor-associated macrophages in colorectal cancer

**DOI:** 10.1038/s41392-020-0124-z

**Published:** 2020-04-15

**Authors:** Xiaojiao Wang, Xi Luo, Chuan Chen, Ye Tang, Lian Li, Banghui Mo, Houjie Liang, Songtao Yu

**Affiliations:** 1 Department of Oncology, Southwest Hospital, Army Medical University, 30 Gaotanyan Street, Chongqing, 400038 People’s Republic of China; 2 Cancer Center, Daping Hospital and Research Institute of Surgery, Army Medical University, Chongqing, 402560 People’s Republic of China; 3 Department of Oncology, Tong Liang District Hospital, Chongqing, 402560 People’s Republic of China

**Keywords:** Cancer microenvironment, Gastrointestinal cancer, Tumour immunology

## Abstract

The inhibitory receptor signal regulatory protein-α (Sirpα) is a myeloid-specific immune checkpoint that engages the “don’t eat me” signal CD47, which is expressed on tumor and normal tissue cells. However, the profile and regulatory mechanism of Sirpα expression in tumor-associated macrophages (TAMs) are still not clear. Here, we found that the expression of Sirpα in TAMs increased dynamically with colorectal cancer (CRC) progression. Mechanistically, CRC cell-derived lactate induced the nuclear translocation of the transcription factor Ap-2α from the cytoplasm in TAMs. Ap-2α functioned as a transcription factor for *Elk-1* by binding to the conserved element GCCTGC located at −1396/−1391 in the mouse *Elk-1* promoter. Subsequently, the Elk-1 protein bound to two conserved sites, CTTCCTACA (located at −229/−221) and CTTCCTCTC (located at −190/−182), in the mouse *Sirpα* promoter and promoted Sirpα expression in TAMs. Functionally, the macrophage-specific knockout of Ap-2α notably promoted the phagocytic activity of TAMs and suppressed CRC progression, whereas these effects were prevented by the transgenic macrophage-specific expression of Elk-1, which regulated TAM phagocytosis and CRC development in a Sirpα-dependent manner. Furthermore, we showed that Elk-1 expression was positively correlated with Sirpα expression in TAMs and was associated with poor survival in CRC patients. Taken together, our findings revealed a novel mechanism through which CRC evades innate immune surveillance and provided potential targets for macrophage-based immunotherapy for CRC patients.

## Introduction

Colorectal cancer (CRC) is a serious disease that endangers human health.^1^ CRC accounts for 10.2% of all cancers, preceded only by lung cancer and breast cancer. CRC is the second leading cause of death, accounting for 9.2% of all cancer-related deaths.^1^ Malignant progression (distant metastasis, malignant proliferation, etc.) is the leading cause of CRC-related death.^2,3^ However, escape from immune surveillance is an important mechanism underlying the survival and metastasis of cancer cells.^4,5^ Therefore, deciphering the molecular and cellular mechanisms by which CRC evades immune surveillance is an urgent need.

Recent studies have shown that the immune status of the tumor microenvironment is an essential factor affecting tumor progression.^4,6–8^ Both innate and adaptive immunity in the tumor microenvironment have been intensively investigated. In recent years, T-cell-based immunotherapy has shown benefits and provided hope for countless patients.^9,10^ Antitumor therapies based on macrophages and other innate immune cells are also in clinical trials.^11–14^

Macrophages, as the first line of innate immunity, kill pathogens and cancer cells through phagocytosis.^15^ Recent studies have shown that macrophages express signal regulatory protein α (Sirpα), a receptor protein that distinguishes “self” and “non-self”, while normal tissue cells express the “self” ligand protein CD47.^16–18^ When the CD47 protein of normal cells binds to the Sirpα receptor on macrophages, the macrophages sense the “self” signal (also called the “don’t eat me” signal) and stop phagocytosis, preventing the destruction of self-tissues. Conversely, in the absence of CD47 recognition, macrophages will maintain active phagocytosis and subsequently clear pathogens, foreign bodies, and tumor cells.

Unfortunately, recent studies have shown that Sirpα/CD47 signaling is often abnormally enhanced in the tumor microenvironment, leading to decreased phagocytosis by tumor-associated macrophages (TAMs) and subsequent cancer progression.^19,20^ Interestingly, Sirpα/CD47 blockade therapies can also enhance the anticancer effects of anti-PD-1, anti-PD-L1, and anti-CTLA-4 antibody immune checkpoint inhibitors that target T cell activation.^11,17^ Therefore, the blockade of the Sirpα/CD47 axis with specific antibodies has shown great potential in cancer therapy.^19^

In addition to treatment with antibodies, the modulation of upstream factors can suppress the Sirpα/CD47 axis. Some studies have shown how CD47 expression is upregulated in cancer cells,^20,21^ whereas the regulatory mechanism underlying Sirpα expression in TAMs is still not clear. Thus, we aimed to examine the expression profile of Sirpα in TAMs and to further identify the upstream transcription factors of Sirpα. We also validated the regulatory roles of these factors in CRC progression. Our findings might help to develop macrophage-based CRC immunotherapy.

## Results

### Sirpα expression is increased in TAMs and positively correlates with CRC progression

**Fig. 1:**
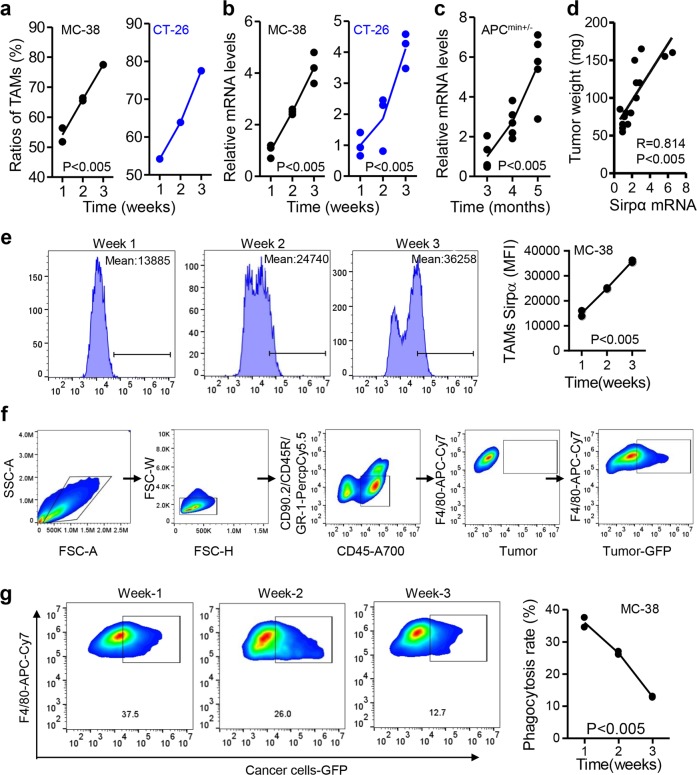
Sirpα expression in tumor-associated macrophages (TAMs) increases with tumor progression. **a** TAMs increased with tumor progression in MC-38 and CT-26 xenograft models. MC-38 or CT-26 cells (1.0 × 10^6^/100 μl of PBS per mouse) were subcutaneously inoculated into 6-week-old WT C57BL/6 or BALB/c mice. TAMs (CD90.2-CD45R-Gr1-CD45+F4/80+) were assessed dynamically by flow cytometry. **b** Sirpα mRNA levels in TAMs increased with tumor progression. The TAMs described above in (**a**) were isolated and subjected to real-time PCR assays to detect Sirpα mRNA levels (*n* = 3). **c** Real-time PCR assays of Sirpα mRNA levels in TAMs from APC^min+/−^ mice were performed at the indicated time points (*n* = 5). **d** Correlation analysis of tumor weight and TAM Sirpα mRNA levels in APC^min+/−^ mice was performed (Pearson *R* = 0.814, *P* < 0.005, *n* = 15). **e** Sirpα protein levels in TAMs from MC-38-based subcutaneous xenograft models were assessed by flow cytometry. **f** The gating strategy for the analysis of in vivo phagocytosis rates with flow cytometry is shown. **g** Phagocytosis rates decreased with tumor growth. GFP-tagged MC-38 cells (1.0 × 10^6^/100 μl PBS) were subcutaneously injected into 6-week-old WT C57BL/6 mice. Phagocytizing macrophages were defined as CD45 + F4/80+GFP+ cells. Phagocytosis rates were measured dynamically with flow cytometry. In (**a**, **b**, **e**, **g**), each sample was pooled from 3 individuals. All data are presented as the mean ± s.e.m. The data in (**a**–**c**, **e**, **g**) were analyzed using one-way ANOVA

To investigate the relationship between macrophage Sirpα and CRC progression, MC-38 cell- and CT-26 cell-based subcutaneous CRC models were employed. In these xenograft models, we showed that the number of infiltrating TAMs increased with tumor progression (Fig. 1a and Fig. S1a). The mRNA levels of Sirpα in TAMs from different tumor models increased dynamically with tumor growth (Fig. 1b, c). In particular, the mRNA levels of Sirpα in TAMs were positively correlated with the weight of spontaneous adenomas in APC^min+/-^ mice (Fig. 1d). Using flow cytometry analysis, we confirmed that the protein levels of Sirpα in TAMs dynamically increased with tumor progression in the MC-38- and CT-26-based subcutaneous tumor models (Fig. 1e and Fig. S1a–c). Given that Sirpα is an inhibitory checkpoint molecule in phagocytosis,^16,22^ we next evaluated the phagocytic activity of TAMs in the context of tumor development. As expected, the in vivo tumor-phagocytosing activity of TAMs declined with tumor growth in the MC-38- and CT-26-based subcutaneous xenograft models (Fig. 1f, g and Fig. S1d).

### Elk-1 is a transcription factor for *Sirpα* in macrophages

Given the close correlation between TAM Sirpα and CRC progression, we further explored the transcription factors that regulate this gene. Functional transcription factors are usually conserved between humans and mice.^23^ Thus, we compared the promoter regions of the human and mouse *Sirpα* genes using online software (https://blast.ncbi.nlm.nih.gov/Blast.cgi) (Fig. S2a). We selected a highly conserved sequence and predicted the binding elements of potential transcription factors with another online tool (http://alggen.lsi.upc.es/). A series of transcription factors (c-Ets-1, Elk-1, C/EBPbeta, YY1, TFII-1, GR-beta, GR-alpha, c-Ets-2, TFIID, and GR) obtained high scores and were considered candidates (Fig. S2b). We silenced the aforementioned factors, which are exclusively expressed in humans and mice. We found that knocking down Elk-1 or TFIID expression obviously attenuated Sirpα mRNA levels in RAW cells (Fig. S2c). TFIID, a universal transcription factor, has been thoroughly explored previously.^24,25^ We found that the expression of TFIID was not associated with tumor progression in the MC-38 cell-based subcutaneous tumor model (Fig. S2d). Thus, we excluded this factor from further analyses. We next focused on Elk-1, which might be a novel transcription factor for Sirpα.

**Fig. 2:**
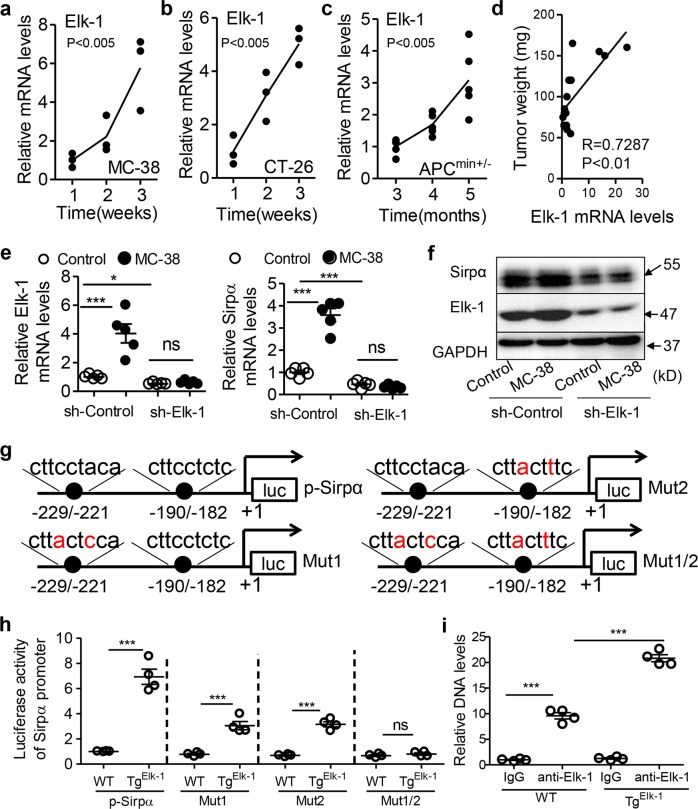
Elk-1 is a transcription factor for *Sirpα* in macrophages. **a**–**c** Elk-1 mRNA levels in TAMs increased with tumor progression in MC-38-based subcutaneous xenograft models (**a**), CT-26-based subcutaneous xenograft models (**b**) and APC^min+/−^ mice at the indicated time points (**c**) (*n* = 3–5). **d** Correlation analysis of tumor weight and TAM Elk-1 mRNA levels in APC^min+/−^ mice was performed (Pearson *R* = 0.7287, *P* < 0.01, *n* = 15). **e** RAW cells were transfected with a scrambled shRNA as a control (sh-Control) or an shRNA specifically targeting mouse Elk-1 (sh-Elk-1) and then treated with MC-38 conditioned medium or normal medium as a control for 24 h. The mRNA levels of Elk-1 and Sirpα in these cells were measured with real-time PCR (*n* = 5). **f** Immunoblotting assays were used to analyze Sirpα expression in the RAW cells described above in (**e**). **g** Mutations in potential Elk-1 binding elements in the mouse Sirpα promoter region are shown. The reporter plasmid p-Sirpα contained a 500-bp wild-type sequence of the mouse Sirpα promoter (−500/+1). Two potential binding sites for the Elk-1 protein were located at −229/−221 and −190/−182, as indicated. Mut-1 contained a mutant Elk-1 binding site at −229/−221. Mut-2 contained a mutant Elk-1 binding site at −190/−182. Mut1/2 contained the mutant sites at both −229/−221 and −190/−182. **h** Luciferase activity of the Sirpα promoter was evaluated. The reporter plasmids described in (**g**) were transfected into peritoneal macrophages from WT or Tg^Elk-1^ mice. Relative luciferase activity was measured (*n* = 4). **i** ChIP assays evaluated the binding activity between the Elk-1 protein and Sirpα DNA. WT and Tg^Elk-1^ peritoneal macrophages were collected and subjected to ChIP assays (*n* = 4). All data are presented as the mean ± s.e.m. The data in (**a**–**c**) were analyzed with one-way ANOVA (*P* < 0.005). The data in (**e**, **h**, **i**) were analyzed with a Student’s *t*-test (***P < 0.005; ns, not significant)

Consistent with the expression profile of Sirpα, the mRNA levels of Elk-1 in TAMs increased with tumor progression in MC-38- and CT-26 cell-based subcutaneous tumor models and in spontaneous tumor models (Fig. 2a-c). We confirmed that the levels of TAM Sirpα were positively correlated with the weight of adenomas in APC^min+/−^ mice (Fig. 2d). We further showed that conditioned medium (CM) from MC-38 cells induced mRNA expression of Elk-1 and Sirpα in RAW cells, whereas silencing Elk-1 diminished these effects (Fig. 2e). In line with the mRNA level data, MC-38 CM-induced Sirpα protein expression was prevented by knocking down Elk-1 expression in macrophages (Fig. 2f).

**Fig. 3:**
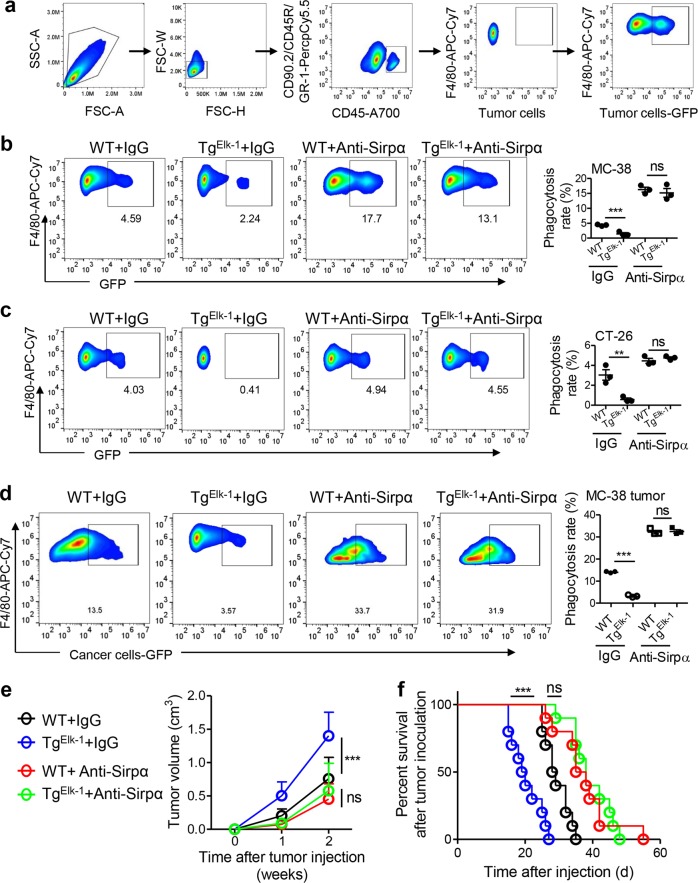
Elk-1-Sirpα regulates phagocytosis and CRC progression. **a** The FACS gating strategy for in vitro tumor phagocytosis is shown. Macrophages were defined as CD45+F4/80+ cells. MC-38 or CT-26 colorectal cancer cells were GFP+. **b** Macrophage Elk-1 suppressed MC-38 phagocytosis in a Sirpα-dependent manner (*n* = 4). **c** Macrophage Elk-1 suppressed Sirpα-dependent CT-26 phagocytosis (*n* = 4). **d** Macrophage Elk-1 suppressed Sirpα-dependent MC-38 phagocytosis in vivo. GFP-tagged MC-38 cells (1.0 × 10^6^/100 μl of PBS) were subcutaneously inoculated into 6-week-old WT or Tg^Elk-1^ mice. Each mouse was treated (i.v.) with anti-Sirpα antibodies or IgG as a control on days 1, 3, and 5 at a dosage of 100 μg/day. Two weeks later, tumors were collected, and phagocytosis rates were measured (*n* = 3). **e** Mice were treated as described in (**d**), and tumor volume was recorded dynamically (*n* = 10). **f** The survival of mice treated as in (**d**) is shown (n = 10, ***P < 0.005; ns, not significant; Gehan–Breslow–Wilcoxon test). The data in **b**–**e** are shown as the mean ± s.e.m (**P < 0.01; ***P < 0.005; ns, not significant; Student’s *t*-test)

To further validate that Elk-1 is a transcription factor for Sirpα, we constructed a luciferase reporter gene under the control of the mouse *Sirpα* promoter. We predicted two potential Elk-1 binding sites located at −229/−221 and −190/−182 upstream of the transcriptional start site in the mouse *Sirpα* gene (Fig. 2g). To observe the function of each site, these sites were mutated individually or simultaneously (Fig. 2g). By using luciferase reporter gene assays, we demonstrated that the transgenic expression of Elk-1 notably increased Sirpα promoter activity in macrophages. This effect was partly attenuated by the mutation of either individual site and was fully prevented by the simultaneous mutation of both sites (Fig. 2h). Chromatin immunoprecipitation (ChIP) assays confirmed the binding of the Elk-1 protein and Sirpα DNA at the aforementioned binding sites (Fig. 2i). The specific transgene expression of Elk-1 in macrophages potentiated this binding activity in peritoneal macrophages (Fig. 2i and Fig. 3a, b). In mouse TAMs, we demonstrated that the binding of the Elk-1 protein and Sirpα DNA increased with tumor progression (Fig. S3c). In human CRC patient samples, we confirmed that the Elk-1 protein could bind to the Sirpα DNA promoter in TAMs and that carcinomas could potentiate this effect (Fig. S3d).

### The Elk-1/Sirpα axis regulates phagocytosis and CRC progression

We next examined whether the Elk-1/Sirpα axis in macrophages regulates phagocytosis and CRC progression because Sirpα functions as an inhibitory checkpoint molecule in tumor phagocytosis.^16^ In vitro phagocytosis assays showed that Elk-1 transgene expression in macrophages notably inhibited the phagocytosis of MC-38 cells and CT-26 cells by macrophages, whereas the blockade of Sirpα with antibodies prevented this activity (Fig. 3a–c). In vivo phagocytosis assays with MC-38 cell-based subcutaneous models yielded similar results (Fig. 3d). Functionally, we showed that the transgenic expression of Elk-1 in macrophages obviously enhanced Sirpα (Fig. S3e) expression, potentiated tumor growth (Fig. 3e) and shortened survival (Fig. 3f). However, treatment with anti-Sirpα antibodies diminished these effects (Fig. 3e, f). These results indicated that macrophage Elk-1 regulated phagocytosis and CRC progression in a Sirpα-dependent manner.

### Ap-2α promotes Elk-1 expression at the transcription level

**Fig. 4:**
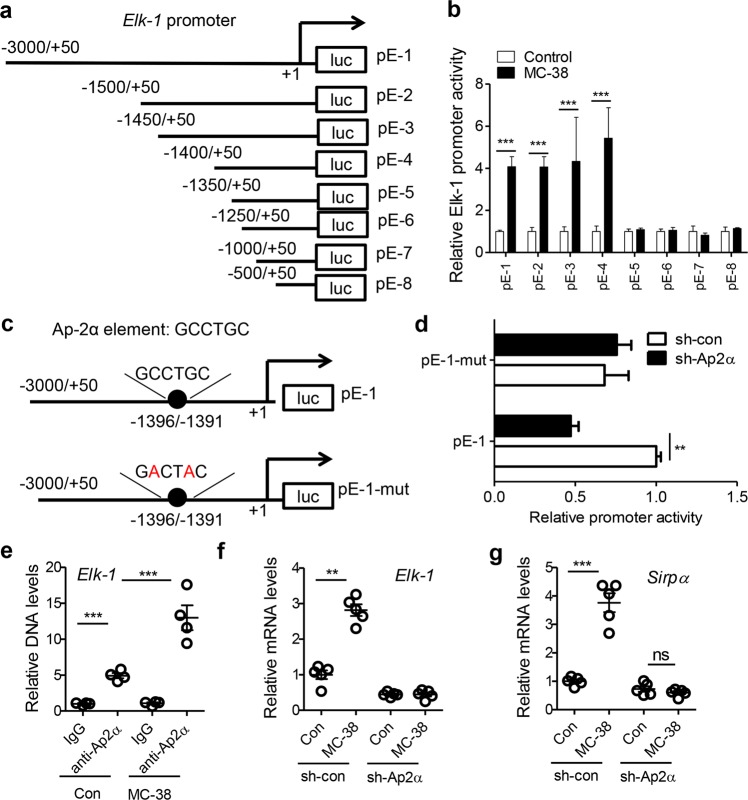
Ap-2α transcriptionally promotes Elk-1 expression. **a** A schematic depiction of different mouse Elk-1 promoter regions that were cloned into the pGL4-basic vector. The constructs were designed as pE-1~8 with different lengths, as indicated. **b** Effects of MC-38 conditioned medium on the promoter activity of the different Elk-1 promoter constructs described in (**a**). Raw cells were transfected with the truncated reporter constructs, stimulated with MC-38 conditioned medium or control normal medium for 24 h, and then harvested for assays assessing luciferase activity. The results are expressed as the relative activity normalized to the activity of each control medium-treated group, which was arbitrarily defined as 1 (*n* = 3). **c** Mutation of a potential Ap-2α binding site in the reporter construct pE-1. The sequence GCCTGC located at −1396/−1391 was mutated to GACTAC in the mutant reporter construct pE-1-mut. **d** Effects of Ap-2α on the activity of the Elk-1 promoter constructs pE-1 and pE-1-mut. RAW cells were cotransfected with pE-1 or Mut-1 plus a scrambled shRNA (sh-con) or an Ap-2α-specific shRNA (sh-Ap2α) for 24 h and then harvested for luciferase activity assays (*n* = 3). **e** Binding activity between Ap-2α and *Elk-1* DNA in normal medium- or MC-38 conditioned medium-stimulated macrophages, as measured by ChIP assays (*n* = 4). **f**, **g** Stimulation of the Ap-2α-dependent mRNA expression of Elk-1 and Sirpα by MC-38 cells. RAW cells were transfected with sh-con or sh-Ap2α for 12 h and then treated with MC-38 conditioned medium or control medium for 24 h. Cells were harvested for real-time PCR analysis of Elk-1 (**f**) and Sirpα (**g**). The data in (**b**, **d**–**g**) are shown as the mean ± s.e.m. (**P < 0.01, ***P < 0.005; ns, not significant; Student’s *t*-test)

Given the important roles of the Elk-1/Sirpα axis in regulating phagocytosis and CRC progression, we next investigated how Elk-1 is regulated in TAMs. We focused on the transcriptional mechanism because the mRNA levels of Elk-1 in TAMs were clearly altered with CRC progression (Fig. 2a-c). We constructed a series of truncated reporter plasmids containing the mouse *Elk-1* gene promoter (Fig. 4a). Reporter gene assays showed that CM from MC-38 cells markedly stimulated mouse Elk-1 promoter activity, and this effect relied on the promoter region −1400/−1350 (Fig. 4b). The DNA sequence located at −1400/−1350 was then used for transcription factor prediction (Fig. S4a, b). The factors expressed exclusively in humans and mice were further silenced with shRNAs in RAW cells. We demonstrated that knocking down the expression of Ap-2α but not the expression of other transcription factors suppressed Elk-1 mRNA levels (Fig. S4c). We predicted a potential Ap-2α binding element, GCCTGC, located at −1396/−1391 in the mouse *Elk-1* gene promoter and −1388/−1383 in the human *ELK-1* gene promoter (Fig. S4d). After the mutation of this element in the mouse *Elk-1* gene promoter, the inhibitory effect of sh-Ap2α on Elk-1 promoter activity disappeared completely (Fig. 4c, d). ChIP assays confirmed that the Ap-2α protein bound to the site located at −1396/−1391 in the mouse *Elk-1* gene promoter and that this binding activity was enhanced by CM from MC-38 cells (Fig. 4e). In MC-38-based xenograft models, we confirmed that the binding activity of the Ap-2α protein and *Elk-1* gene promoter in TAMs increased with tumor progression (Fig. S4e). This binding activity in TAMs from human carcinoma tissue samples was stronger than that in TAMs from adjacent normal tissue samples (Fig. S4f). Furthermore, we showed that silencing Ap-2α with shRNAs prevented the MC-38 CM-induced mRNA expression of Elk-1 (Fig. 4f) and Sirpα (Fig. 4g).

### The macrophage Ap2α-Elk-1 axis regulates CRC progression

**Fig. 5:**
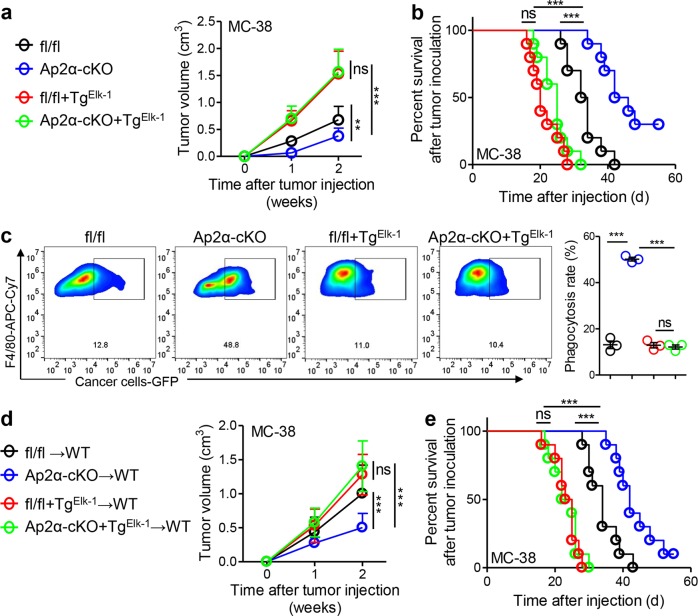
Macrophage Ap2α-Elk-1 axis regulates CRC progression. **a** MC-38 cells (1.0 × 10^6^ cells per mouse) were subcutaneously inoculated into fl/fl, Ap2α-cKO, fl/fl + Tg^Elk-1^, and Ap2α-cKO + Tg^Elk-1^ mice, and tumor volume was monitored dynamically (*n* = 10). **b** The survival of the mice described above in (**a**) is shown (*n* = 10, ***P < 0.005; ns, not significant; Gehan–Breslow–Wilcoxon test). **c** The Elk-1 transgene prevented Ap-2α silencing-induced tumor phagocytosis. Six-week-old male fl/fl, Ap2α-cKO, fl/fl + Tg^Elk-1^, and Ap2α-cKO + Tg^Elk-1^ mice were subcutaneously engrafted with GFP-tagged MC-38 cells (1.0 × 10^6^ cells per mouse). Two weeks later, tumors were recovered for the measurement of tumor phagocytosis by TAMs (*n* = 3). **d** Volumes of tumors in WT mice inoculated with MC-38 cells mixed 2:1 with macrophages isolated from corresponding tumors grown in fl/fl, Ap2α-cKO, fl/fl + Tg^Elk-1^ or Ap2α-cKO + Tg^Elk-1^ mice are shown (*n* = 10). **e** The survival of the mice described above in (**d**) is shown (*n* = 10, ***P < 0.005; ns, not significant; Gehan–Breslow–Wilcoxon test). The data in (**a**, **c**, **d**) are shown as the mean ± s.e.m. (**P < 0.01, ***P < 0.005; ns, not significant; Student’s *t*-test)

To investigate the function of the macrophage Ap2α/Elk-1 axis in CRC progression, we established a mouse model with the myeloid-specific knockout of the *Ap2α* gene (Ap2α-cKO) (Fig. S5a). These mice were crossed with transgenic mice carrying a myeloid-specific transgene encoding *Elk-1* (Tg^Elk-1^). MC-38 cell-based subcutaneous models were established in these genetically modified mice. We showed that Ap2α-cKO mice exhibited downregulated Elk-1 and Sirpα expression, whereas Tg^Elk-1^ mice showed rescued expression in TAMs (Fig. S5b). Functionally, we found that subcutaneous tumors in Ap2α-cKO mice grew much slower than those in control littermates (Fig. 5a). The inhibitory effects on CRC growth observed in Ap2α-cKO mice disappeared in Tg^Elk-1^ mice (Fig. 5a). In line with the tumor growth results, Ap2α-cKO mice exhibited prolonged survival, but this improvement was prevented in Tg^Elk-1^ mice (Fig. 5b). In vivo phagocytosis assays showed that the myeloid-specific ablation of Ap2α induced tumor phagocytosis, and this effect was fully prevented by the expression of the Elk-1 transgene in macrophages (Fig. 5c). To further verify the role of the macrophage Ap2α/Elk-1 axis in CRC progression, we isolated TAMs from different genetically modified mice and performed adoptive therapy in WT mice. We validated that Ap2α-cKO-suppressed Sirpα expression was abolished by the expression of the Elk-1 transgene in macrophages (Fig. S5c). We showed that Ap2α-cKO TAMs suppressed tumor growth, whereas the addition of TAMs from Tg^Elk-1^ prevented this effect (Fig. 5d). Consistent conclusions were obtained when observing the survival of tumor-bearing mice undergoing adoptive therapy (Fig. 5e).

### Lactate induces TAM Ap-2α activity and CRC progression

Given the continuous increase in TAM Elk-1 expression with CRC progression (Fig. 2a-c), we examined whether Ap-2α expression increases dynamically. Unexpectedly, the mRNA levels of Ap-2α in TAMs remained unchanged with CRC progression in MC-38 cell- and CT-26 cell-based subcutaneous tumor models and in APC^min+/−^ mice (Fig. S6a–c). In vitro tests showed that CM from MC-38 or CT-26 cancer cells did not induce Ap-2α expression at the mRNA (Fig. S6d) or protein level (Fig. S6e). However, both MC-38 CM and CT-26 CM stimulated the activity of the Elk-1 promoter in reporter gene assays (Fig. S6f). These findings indicated that CM from cancer cells might stimulate Ap-2α activity but not Ap-2α expression.

**Fig. 6:**
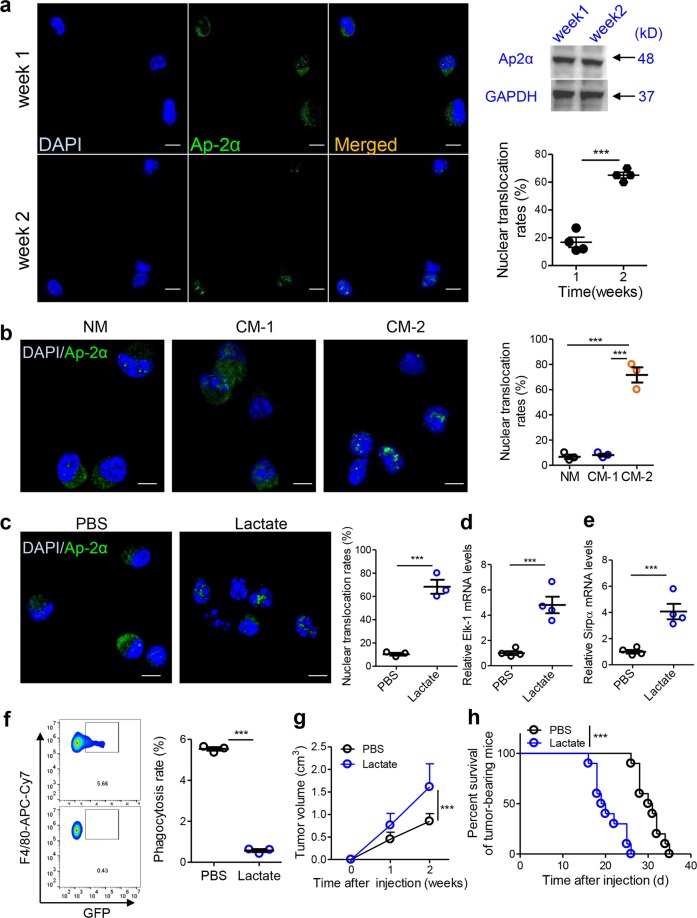
Lactate induces TAM Ap-2α activity and CRC progression. **a** Nuclear translocation of Ap-2α in TAMs was evaluated. Six-week-old C57BL/6 mice were subcutaneously inoculated with MC-38 cells (1.0 × 10^6^ cells per mouse). TAMs isolated at different time points were stained with anti-Ap-2α antibodies (green). Nuclei were visualized by DAPI staining (blue). The total Ap-2α protein in TAMs at different time points was measured by western blotting assays. The nuclear translocation rates of Ap-2α were calculated (*n* = 4). **b** Metabolic factors induced the nuclear translocation of Ap-2α in macrophages. MC-38 conditioned medium was roughly fractionated into a protein fraction (CM-1, >3 kD) and a metabolic fraction (CM-2, <3 kD) by molecular size. Bone marrow-derived macrophages were treated with CM-1, CM-2 or control normal medium for 24 h and then stained with anti-Ap-2α antibodies as described in (**a**) (*n* = 3). **c** Lactate stimulated the nuclear translocation of Ap-2α in macrophages. Bone marrow-derived macrophages were treated with lactate (10 mM) or PBS as a control for 24 h. Then, cells were stained with anti-Ap-2α antibodies as described in (**a**) (*n* = 3). **d**, **e** The mRNA levels of Elk-1 (**d**) and Sirpα (**e**) in macrophages treated with lactate (10 mM) or PBS for 24 h were measured (*n* = 4). **f** The phagocytosis rates of macrophages treated with lactate (10 mM) or PBS for 24 h were evaluated (*n* = 3). **g** Six-week-old male C57BL/6 mice were subcutaneously engrafted with MC-38 cells (1.0 × 10^6^ cells per mouse) and then subcutaneously treated with lactate (50 μmol in 100 μl of PBS per injection) or PBS on days 1, 3, and 5. Tumor volume was measured dynamically (*n* = 10). **h** The survival of the mice described in (**g**) is shown (*n* = 10, ***P < 0.005; Gehan–Breslow–Wilcoxon test). The data in (**a**–**g**) are reported as the mean ± s.e.m. (***P < 0.005; Student’s *t*-test)

Nuclear translocation is positively correlated with transcription factor activity.^26,27^ As expected, we showed that the nuclear translocation (but not the total protein) of Ap-2α in TAMs increased with tumor progression in MC-38 cell-based xenograft models (Fig. 6a). To determine which factors from CRC cells can induce the translocation of Ap-2α in TAMs, we fractionated MC-38 CM into metabolic (<3 kD) and protein fractions (>3 kD). We found that cancer cell-derived metabolic factors stimulated the nuclear translocation of Ap-2α in bone marrow-derived macrophages (BMDMs) (Fig. 6b). Lactate is one of the most well-known and abundant metabolites released by cancer cells. Thus, we next validated the stimulatory role of lactate in Ap-2α translocation. The results showed that lactate obviously induced Ap-2α translocation from the cytoplasm to the nucleus (Fig. 6c). Notably, this effect was abolished with Ldha knockdown (Fig. S6g). This result indicated that lactate itself, but not the low-pH environment it induced, promoted the translocation of Ap-2α.

We further demonstrated that lactate-induced changes in mRNA levels of Elk-1 and Sirpα were prevented by Elk-1 silencing in peritoneal macrophages (Fig. 6d, e and Fig. S6h, i). The lactate-induced nuclear translocation of Ap-2α, as well as changes in expression levels of Elk-1 and Sirpα, were prevented by Ap-2α knockout in peritoneal macrophages (Fig. S6j–l). Functionally, lactate treatment obviously suppressed phagocytosis rates, potentiated tumor growth and shortened survival in mice bearing subcutaneous MC-38 tumors (Fig. 6f–h). Notably, the effects of lactate on the phagocytosis and growth of MC-38 tumors were dependent on Ap-2α expression in macrophages (Fig. S6m, n).

### TAM Elk-1 and Sirpα levels increase with CRC progression

**Fig. 7:**
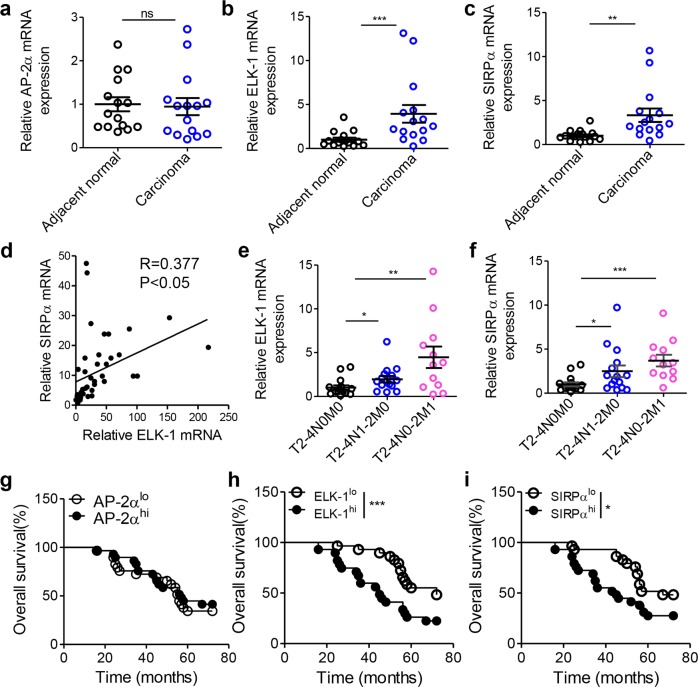
Macrophage Elk-1 and Sirpα levels increase with CRC progression. **a**–**c** mRNA levels of Ap-2α (**a**), Elk-1 (**b**) and Sirpα (**c**) in macrophages from adjacent normal and carcinoma tissue samples from CRC patients (*n* = 15). **d** Positive correlation of **S**irpα expression with Elk-1 expression in macrophages from each individual sample. Linear regression was performed (*n* = 42, *P* < 0.05, Pearson *R* = 0.377). **e**, **f** mRNA levels of Elk-1 and Sirpα in TAMs from CRC patients with different TNM stages. Samples were divided into 3 groups according to the TNM stage: T2-4N0M0 (*n* = 15), T2-4N1-2M0 (*n* = 15) and T2-4N0-2M1 (*n* = 12). **g**–**i** Overall survival of CRC patients with differential expression of AP-2α (**g**), ELK-1 (**h**) and SIRPα (**i**). Patients were placed into two groups according to the mRNA expression of AP-2α, ELK-1 or SIRPα in TAMs. Patients with expression levels in the top 50% for TAM AP-2α, ELK-1 or SIRPα expression were assigned to the AP-2α^hi^, ELK-1^hi^ or SIRPα^hi^ groups, respectively. The remaining patients with low TAM AP-2α, ELK-1 or SIRPα expression were assigned to the AP-2α^lo^, ELK-1^lo^ or SIRPα^lo^ groups, respectively. The overall survival time of patients was determined during follow-up visits (*n* = 58, *P < 0.05, ***P < 0.005, Gehan–Breslow–Wilcoxon test). The data in (**a**–**c** and **e**, **f**) represent the mean ± s.e.m. (*P < 0.05, **P < 0.01 and ***P < 0.005; Student’s *t*-test)

To correlate the aforementioned results with physiopathology in the clinic, we measured the expression of AP-2α, ELK-1, and SIRPα in macrophages from adjacent normal or carcinoma tissue samples. We found that the mRNA levels of macrophage AP-2α were not different in adjacent normal and carcinoma tissue samples (Fig. 7a). However, TAMs in carcinoma tissues had obviously higher translocation rates of AP-2α than those from adjacent normal tissues (Fig. S7a, b). The mRNA and protein levels of macrophage ELK-1 (Fig. 7b and Fig. S7a, c) and SIRPα (Fig. 7c and Fig. S7a, d) in carcinoma tissue samples were much higher than those in adjacent normal tissue samples. A correlation study showed that macrophage ELK-1 mRNA levels were positively correlated with macrophage SIRPα mRNA levels (Fig. 7d). In addition, we demonstrated that the mRNA expression of TAM ELK-1 and SIRPα was obviously higher in the metastatic stage than in the early phase (Fig. 7e, f). Consistently, increased levels of ELK-1 or SIRPα in TAMs predicted poor survival in CRC patients (Fig. 7g–i).

## Discussion

Inhibitory immune checkpoint blockade has been one of the most significant advances in anticancer therapy in the past decade.^28,29^ To date, research has mainly focused on improving adaptive immune functions, but recent studies have indicated that the Sirpα-CD47 pathway, a phagocytosis checkpoint in macrophages and other innate immune cells, may be an interesting therapeutic target.^17,19,22^ Recent strategies for the blockade of the Sirpα-CD47 axis mainly rely on antibodies.^13,17^ Here, we focused on the regulatory mechanism underlying SIRPα expression and identified two upstream transcription factors, Ap-2α and ELK-1. These factors might serve as potential therapeutic targets in CRC patients.

Studies regarding the regulatory mechanisms of Sirpα expression are rather limited. Kong X et al. reported that the LPS-induced downregulation of Sirpα contributed to innate immune activation in macrophages.^19^ Lin Y et al.claimed that notch signaling modulated macrophage polarization and phagocytosis through the suppression of Sirpα.^30^ Zhu D et al. reported that Sirpα was directly targeted by microRNA-17/20a/106 and was involved in macrophage inflammatory responses. However, whether these mechanisms exist in tumors is still unclear.^20^ Our study first uncovered a clear mechanism linking the tumor microenvironment to Sirpα expression, phagocytosis deficiencies and cancer progression.

Our study provides a new strategy to indirectly block the Sirpα-CD47 pathway. In addition to Sirpα in TAMs, CD47 in tumor cells can also be suppressed by other factors. It has been reported that MYC is a transcription factor for CD47 in cancer cells. Therefore, the inhibition of MYC could also decrease CD47 expression and potentiate phagocytosis by TAMs.^20^ More upstream regulatory factors, especially cancer cell- and macrophage-specific factors, urgently need to be identified. Increased target specificity is expected to reduce the side effects resulting from extensive intervention involving CD47 or Sirpα.

Our findings in the present study might be relevant in other types of cancers. Lactate, which is highly produced by a vast number of cancers,^31^ might induce Ap-2α activity and subsequently promote Elk-1 and Sirpα expression in TAMs. Our results showed that the binding elements of Ap-2α and Elk-1 were highly conserved between mice and humans, indicating a common regulatory mechanism underlying the Ap-2α/Elk-1/Sirpα axis. However, this finding needs to be validated in future studies.

Lactate has long been known as a negative regulator of antitumor immunity.^31,32^ For example, lactate can induce TAMs to differentiate into an M2-like phenotype by activating HIF-1α, thus promoting tumor development.^31^ Here, we found that lactate could stimulate the translocation of Ap-2α from the cytoplasm to the nucleus, thus potentiating the Elk-1/Sirpα axis-mediated “don’t eat me” signal. It should be noted that the detailed mechanism linking lactate to Ap-2α activation is still obscure. An alteration in the pH value or metabolic pathways might be involved in this process since a variety of transcription factors are regulated by lactate in these ways.^31^

**Fig. 8:**
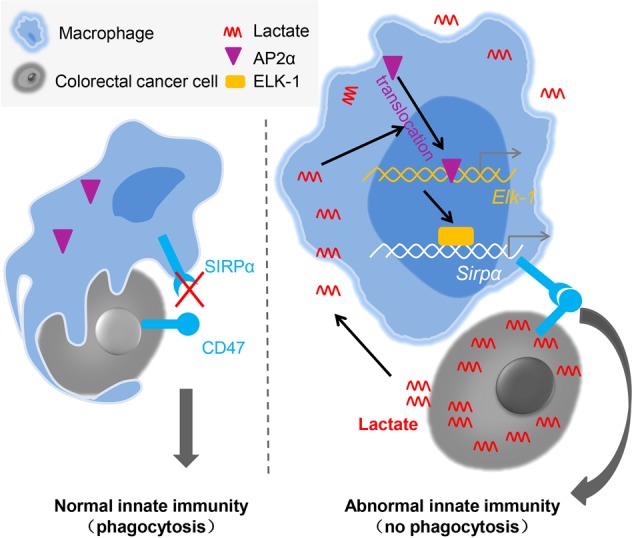
The working model for CRC evasion of innate immune surveillance. Without a cancer challenge, the interaction between macrophage SIRPα and CRC CD47 is interrupted, and macrophages exhibit normal phagocytic function. In the CRC microenvironment, CRC-derived lactate induces the activity of the transcription factor Ap-2α and the expression of its target Elk-1, which further promotes the expression of Sirpα in TAMs. The enhanced interaction between macrophage SIRPα and CRC CD47 reduces the phagocytic function of TAMs

Collectively, our results demonstrated that CRC-derived lactate induced the activity of the transcription factor Ap-2α and the expression of its target Elk-1, which further promoted the expression of the inhibitory immune checkpoint receptor Sirpα (Fig. 8). Our findings revealed a molecular and cellular mechanism by which CRC evades innate immune surveillance. Related factors might serve as therapeutic targets in CRC patients.

## Materials and methods

### Cell culture

The mouse colorectal cancer cell lines MC-38 (JENNIO, Guangzhou, China) and CT-26 (American Type Culture Collection (ATCC), MD, USA) and the macrophage-like cell line Raw264.7 (RAW cells) (ATCC, MD, USA) were maintained in our lab. All cells were authenticated and tested for mycoplasma. Primary macrophages and all cell lines were cultured in DMEM or RPMI 1640 medium supplemented with 10% fetal bovine serum (FBS) at 37 °C in a humidified 5% CO_2_ atmosphere.

### Mice

Mouse studies were approved by the Institutional Animal Care and Use Committee of Third Military Medical University (Army Medical University) and were carried out according to relevant guidelines. All the mice were maintained in a specific pathogen-free environment. Myeloid-specific knockout and transgenic mouse models involving the lysozyme or CD11b promoter are commonly used to investigate the in vivo functions of specific genes in macrophages. A cDNA sequence encoding mouse *Elk-1* was subcloned into a transgenic construct containing the human CD11b promoter to drive myeloid-specific gene expression. The myeloid-specific *Elk-1* transgene construct was microinjected into C57BL/6 embryos according to standard protocols, and the resulting founders were crossed with wild-type (WT) C57BL/6 mice. The line with the highest expression of Elk-1 in macrophages (Tg^Elk-1^) was selected for further study. Myeloid cell-specific Ap-2α knockout (Ap2α-cKO) mice were obtained by crossing Ap-2α-floxed mice (#023406, Jackson Laboratory Stock) with mice expressing lysozyme promoter-driven Cre recombinase (#004781, Jackson Laboratory Stock). To restore the expression of Elk-1 in myeloid cells, Ap2α-cKO mice were crossed with Tg^Elk-1^ mice. APC^min+/-^ mice that develop spontaneous adenomas were purchased from the Jackson Laboratory (Stock #002020).

### shRNA-mediated gene silencing

An shRNA specific to Ap-2α, Elk-1, C/EBPb, YY1, TFIID, C/EBPα, C/EBPδ, STAT4, NF-1 or STAT5A or a scrambled shRNA, which was used as a control, was transfected into macrophages. Twenty-four hours later, cells were harvested for further experiments. Each gene was targeted by two different shRNAs. The shRNA sequences were provided by Sigma-Aldrich (MISSION® shRNA) and are listed in Table S1.

### Preparation of CM

MC-38 cells were cultured in 250-ml flasks in regular medium (DMEM supplemented with 10% FBS). When the cells reached 80% confluence, 10 ml of DMEM with 1% FBS was added to each flask and was collected 24 h later. This collected medium was mixed with regular medium (v/v = 1:1) to generate MC-38 CM. This CM was used to treat macrophages in vitro.

### Isolation of peritoneal macrophages (PMs)

Each mouse was intraperitoneally injected with 2 ml of 3% thioglycollate (#T9032, Sigma) on day 0 and euthanized with CO_2_ on day 2. Peritoneal cells were collected by washing with 5 ml of DMEM supplemented with 1% penicillin and streptomycin. After centrifugation, peritoneal cells were resuspended and cultured in cell culture dishes. One hour later, any floating cells were removed, and the attached cells were considered PMs.

### Isolation of bone marrow-derived macrophages (BMDMs)

The bone marrow medium (BMM) used for BMDM induction was generated by mixing 70 ml of regular DMEM with 30 ml of CM from L929 murine fibroblast cells. A mouse was euthanized with CO_2_ on day 1, and the femur and tibia were harvested for the collection of bone marrow. Bone marrow-derived cells were plated in 6-well plates containing 4 ml of BMM in each well. Fresh BMM (1 ml per well each time) was added to cultured cells on days 3 and 5. The medium was changed to regular medium on day 6, and cells attached to the bottom of the plate were considered BMDMs.

### Isolation of TAMs

Fresh CRC tissue samples were cut into pieces (<1 m^3^) and digested in Buffer A (PBS with 0.5% endotoxin-free BSA and 2 mM EDTA) supplemented with 1 g/L collagenase IV (#LS004188, Worthington), 0.1 g/L hyaluronidase (#H1115000, Sigma) and 0.01 g/L DNase I (#D8071, Solarbio, China). Dissociated cells were collected in a 15-ml tube and centrifuged at 400 × *g* for 5 min. Pellets were resuspended in ACK lysing buffer (#C3702, Beyotime, Shanghai, China) for 5 min and then washed with Buffer A before filtration with a 100-μm filter. These cells were collected to further isolate macrophages (CD45+F4/80+) by fluorescence-activated cell sorting (FACS).

### Fluorescence-activated cell sorting (FACS)

Tumor tissue-derived cells in Buffer A (PBS with 0.5% endotoxin-free BSA and 2 mM EDTA) were counted. An aliquot of cells was pelleted and resuspended in Sorting Buffer (PBS with 0.5% endotoxin-free BSA, 2 mM EDTA and 25 mM HEPES) at 10^7^ cells/ml. Cells were incubated with Fc Block (#553142, BD Biosciences) prior to staining with conjugated antibodies for 15 min at 4 °C and then washed twice in Sorting Buffer. Cells were then resuspended in Sorting Buffer for FACS (FACSAria II or Accuri C6, BD Biosciences). The antibodies included a PerCP/Cy5.5-conjugated anti-mouse Ly-6G/Ly-6C (Gr-1) antibody (#108427, BioLegend), a PerCP/Cy5.5-conjugated anti-mouse CD90.2 antibody (#140321, BioLegend), a PerCP/Cy5.5-conjugated anti-mouse/human CD45R/B220 antibody (#103235, BioLegend), an AF700-conjugated anti-mouse CD45 antibody (#103128, BioLegend), an APC/Cy7-conjugated anti-mouse F4/80 antibody (#123118, BioLegend), a FITC-conjugated anti-mouse CD172a (Sirpα) antibody (#144006, BioLegend), a PerCP/Cy5.5-conjugated anti-human CD45 antibody (#368504, BioLegend) and an APC-conjugated anti-human CD68 antibody (#333809, BioLegend). Mouse TAMs were defined as CD90.2-CD45R-Gr1-CD45+F4/80+ cells. When macrophages were isolated from GFP-tagged MC-38 tumors, GFP+ cells were excluded. Human TAMs were defined as CD45+CD68+ cells.

### Isolation of TAMs from CRC patients

Human TAMs were isolated from CRC patients at PLA 324 Hospital and Southwest Hospital in Chongqing (China). This experiment was carried out with permission from the Institutional Research Ethics Committee of Third Military Medical University. All participants gave informed consent. Tumor tissue samples were collected in compliance with the regulations approved by the Scientific Investigation Board of the hospitals. Forty-two samples of CRC tissue with different TNM stages were collected. Patient information is included in Table S2. All tumors were primary tumors and untreated before surgery, and specimens were anonymized. Macrophages (CD45+CD68+) were isolated with FACS as described above and subjected to RNA extraction and reverse transcription. cDNAs were stored at −80 °C before PCR assays. The investigator who performed the PCR assays was blinded to the patient information. The primers used for AP-2α, ELK-1, and SIRPα are listed in Table S3.

### Subcutaneous tumor models

Six-week-old male C57BL/6 mice (WT or genetically modified) were subcutaneously engrafted with MC-38 cells or GFP-tagged MC-38 cells (1.0 × 10^6^ cells in 100 µl of PBS per mouse) in the thigh. Six-week-old male BALB/c mice were subcutaneously inoculated with CT-26 cells (1.0 × 10^6^ cells in 100 µl of PBS per mouse). Tumor volume was dynamically measured and calculated as 0.523× (length × width × height). This experiment was approved by the Institutional Animal Care and Use Committee of Third Military Medical University and was carried out in accordance with the relevant guidelines.

### In vitro phagocytosis assays

Peritoneal macrophages were plated (5.0 × 10^5^ per well) in a 6-well culture plate and incubated in serum-free medium for 1 h before adding GFP-tagged CT-26 or MC-38 cells (1.0 × 10^6^ per well). After coculturing at 37 °C for 4 h, cells were harvested for flow cytometry. Macrophages were defined as CD45+F4/80+ cells. Phagocytizing macrophages were defined as CD45+F4/80+GFP+ cells.

### In vivo phagocytosis assays

Six-week-old male C57BL/6 mice were subcutaneously engrafted with GFP-tagged MC38 cells (1.0 × 10^6^ cells per mouse) and were euthanized with CO_2_ at different time points. Tumors were collected and subjected to TAM counting by FACS. TAMs were isolated as described above in “Isolation of TAMs”. Phagocytizing macrophages were defined as CD45+F4/80+GFP+ cells.

### In vivo treatment with antibodies

Mice bearing CM-38 tumors were treated with monoclonal anti-Sirpα antibodies (#144002, BioLegend) or control IgG antibodies (#400402, BioLegend). Tumor cells were injected on day 0, and antibodies were administered via the tail vein 3 times (days 1, 3 and 5) at a dosage of 100 μg/day.

### Real-time PCR

Total RNA extracted with a kit (#10296010, Thermo Fisher Scientific) was transcribed into cDNA using PrimeScript (#DRR047A, Takara, Dalian, China). qPCR was carried out using an ABI 7500 Real-Time PCR system (Applied Biosystems, Darmstadt, Germany). Reactions were carried out using Tli TNaseH plus and a universal PCR master mix (#RR820A, TakaRa). The expression level of GAPDH was used as an internal control. Relative expression was calculated by the 2^(−DDCt)^ method. The primers used are listed in Table S3.

### Western blotting assays

Total protein was isolated with RIPA buffer (#P0013, Beyotime, China) and quantified with a BCA kit (#P0009, Beyotime, China). Each sample (50 μg) was separated by 10% SDS-PAGE and transferred to a polyvinylidene difluoride membrane, which was then blocked with 5% BSA and incubated with primary antibodies overnight at 4 °C. Membranes were then rinsed 3 times with PBS containing 0.1% Tween 20 (PBST) and incubated with appropriate secondary antibodies at room temperature for 1 h. Then, membranes were washed with PBST 3 times. Signals were generated with Enhanced Chemiluminescence Substrate (#NEL105001 EA, PerkinElmer) for 1 min before detection with a Bio-Rad ChemiDoc MP System (170-8280). The primary antibodies included anti-Ap-2α (#3215, Cell Signaling; the dilution ratio was 1:1000), anti-Elk-1 (#9182, Cell Signaling; the dilution ratio was 1:1000), anti-Sirpα (#13379, Cell Signaling; the dilution ratio was 1:1000) and anti-GAPDH (#5174, Cell Signaling; the dilution ratio was 1:2000) antibodies. Images were cropped for presentation.

### Immunofluorescence staining

Isolated TAMs or BMDMs on coverslips were fixed in 4% ice-cold paraformaldehyde in PBS for 30 min, washed with PBS 3 times and incubated in a protein-blocking solution at room temperature for 30 min. The cells on coverslips were stained with an anti-Ap-2α antibody (#3215, Cell Signaling, the dilution ratio was 1:500) at 37 °C for 1 h and then 4 °C overnight. After washing, coverslips were incubated at 37 °C for 1 h with a TRITC-conjugated goat anti-rabbit IgG antibody (1:50, Beyotime, China). Cell nuclei were visualized by counterstaining with 4′,6-diamidino-2-phenylindole (DAPI). The specificity of the primary antibody was verified by omitting that antibody in the reaction.

### DNA constructs and reporter gene assays

DNA fragments for the mouse Sirpα and Elk-1 promoter fusion reporter constructs are shown in Fig. 2g, Fig. 4a and c and were generated from the genomic DNA of mouse peritoneal macrophages by PCR amplification. The primers used for the DNA fragments are listed in Table S4. The restriction endonucleases NheI (GCTAGC) and BglII (AGATCT) were selected. Amplified DNA fragments were subcloned into the pGL4-Basic vector. Site-directed mutagenesis was performed using a TakaRa MutanBEST Kit (D401, Takara). Reporter constructs were transfected into RAW cells, and luciferase activities of cell lysates were evaluated according to the manufacturer’s instructions (Promega Corp., Madison, WI). The total protein concentration in each sample was measured as an internal control.

### Chromatin immunoprecipitation (ChIP) assays

ChIP assays were performed to study the interaction between the Elk-1 protein and Sirpα promoter DNA as well as between the AP-2α protein and Elk-1 promoter DNA in primary macrophages, including peritoneal macrophages and TAMs from mice or patients. Briefly, macrophages (1 × 10^6^) were cross-linked with 1% formaldehyde, followed by sonication in a Microson 500-W Ultrasonicator (Sonics & Materials, Inc., Newtown, CT) at 38% of the maximum power for approximately eight to ten consecutive cycles of 10-s pulses. After centrifugation at 18,000 × *g* for 10 min, supernatants with equal amounts of protein were immunoprecipitated with 1 μg of specific antibodies or a control IgG antibody using a ChIP kit (Millipore Corp.) according to the manufacturer’s protocol. Immunoprecipitates were analyzed by real-time PCR to detect any immunoprecipitated DNA harboring functional Elk-1 or AP-2α binding elements in the Sirpα or Elk-1 promoter, respectively. The primers for the Sirpα and Elk-1 promoter DNA sequences are listed in Table S5.

### Statistical analysis

Statistical analyses were performed using GraphPad Prism 5 (GraphPad Software, Inc.). Survival data were analyzed using a Gehan–Breslow–Wilcoxon test. All other data are displayed as the means±standard error of the means (s.e.ms.) and were analyzed using either one-way ANOVA or a two-tailed unpaired Student’s *t*-test. For each parameter of all data presented, * indicates *P* < 0.05, ** indicates *P* < 0.01, and *** indicates *P* < 0.005.

## Supplementary information

Supplementary information

## Data Availability

All the data supporting the findings of this work are available within the article and its Supplementary information files.
