# Supplementary material for: The Ap-2α/Elk-1 axis regulates Sirpα-dependent tumor phagocytosis by tumor-associated macrophages in colorectal cancer
Source: Signal Transduct Target Ther. 2020 Apr 15;5:35. doi: 10.1038/s41392-020-0124-z (PMC7156469; doi:10.1038/s41392-020-0124-z)
Supplement: Supplementary file 1 — Supplementary information [file 41392_2020_124_MOESM1_ESM.docx]

Supplementary Materials for

Ap-2α/Elk-1 axis regulates Sirpα-dependent tumor phagocytosis by tumor-associated macrophages in colorectal cancer

Xiaojiao Wang^1#^, Xi Luo^1#^, Chuan Chen^2#^, Ye Tang^3^, Lian Li^1^, Banghui Mo^1^, Houjie Liang^1*^, Songtao Yu^1*^

Correspondence to: songtaoyu@yahoo.com; lianghoujie@sina.com

**This PDF file includes:**

Figures. S1 to S7

Tables S1 to S5


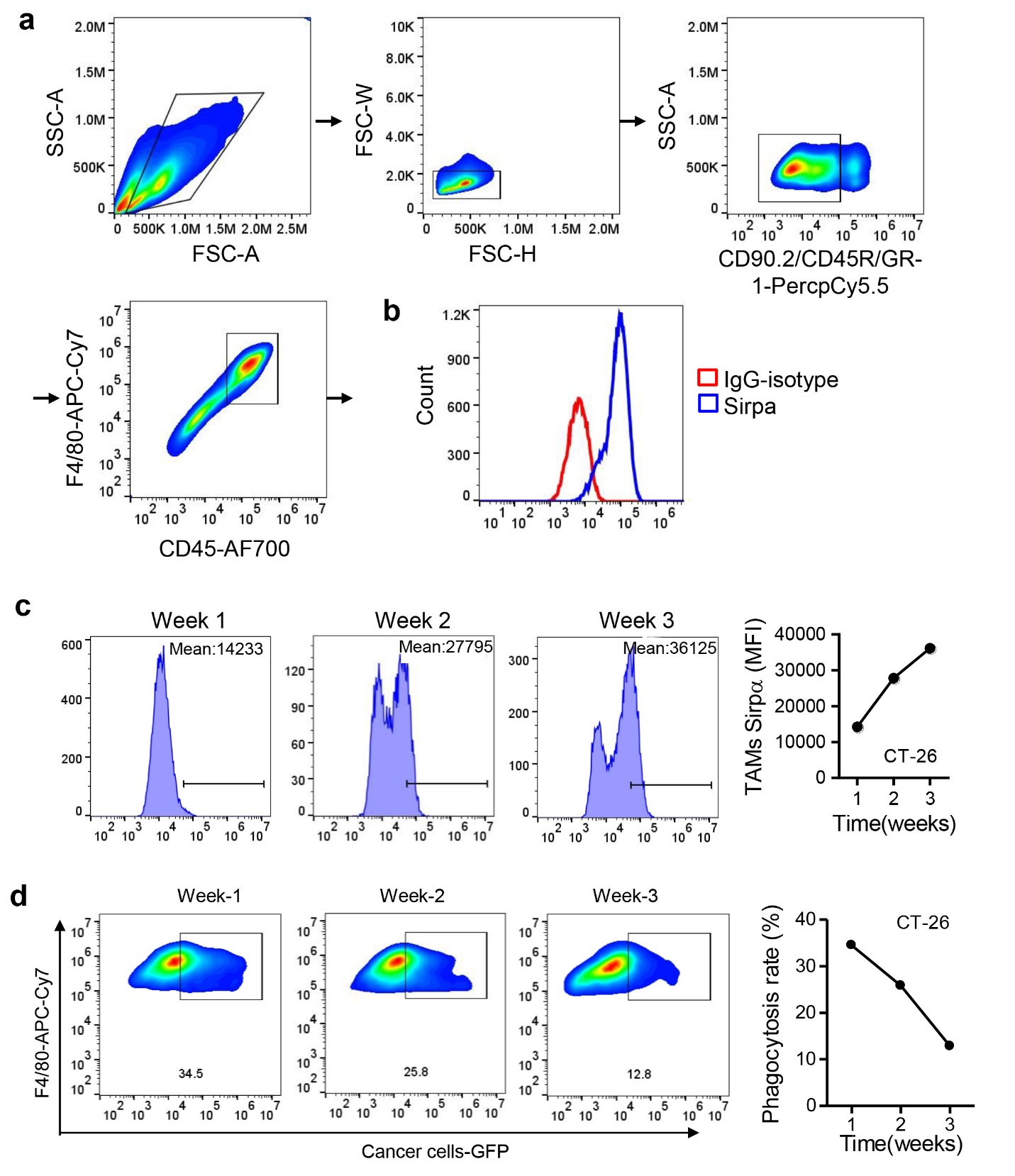


Figure. S1.

**Sirpα in tumor associated macrophages (TAMs) increased with tumor progression**

**(a,b)** FACS gating strategy for the analysis of tumor associated macrophages (TAMs) and Sirpα protein levels. TAMs were marked as CD90.2-CD45R-GR1-CD45+F4/80+.

**(c)** Sirpα protein levels in TAMs increased with tumor growth in CT-26 cell-based subcutaneous xenograft models. Each plot represents a pooled sample from 3 individual ones.

**(d)** Phagocytosis rates were decreased with tumor growth. GFP-tagged CT-26 cells (1.0 × 10^6^/100 μl PBS) were subcutaneously injected in 6-week-old WT BALB/c mice. Phagocytic macrophages were assessed as CD45+F4/80+GFP+. Phagocytosis rates were measured dynamically with flow cytometry. Each plot represents a pooled sample from 3 individual ones.


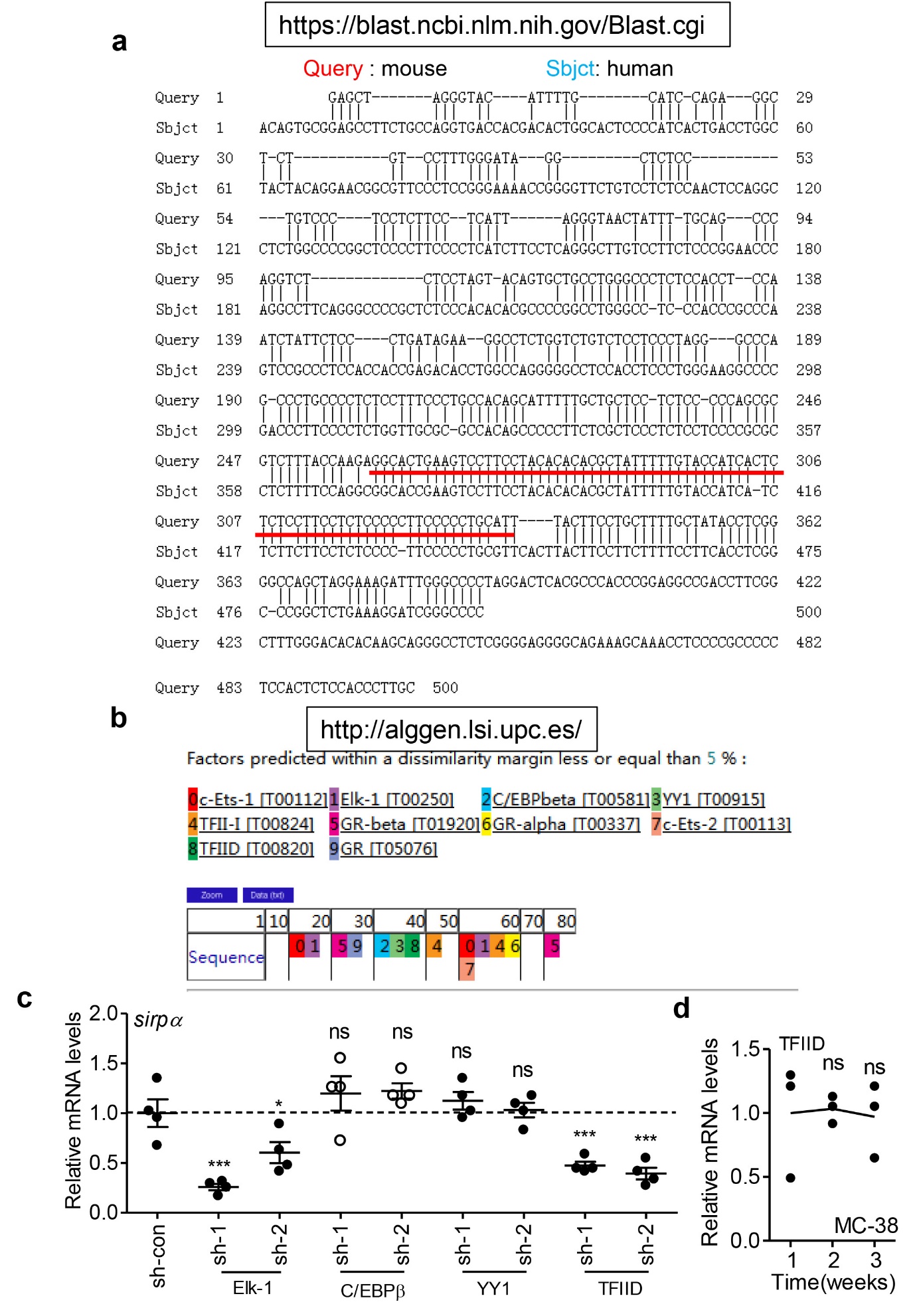


**Figure. S2.**

**Prediction of transcriptional factor for gene *sirpα***

**(a)** Alignment of human and mouse *Sirpα* gene promoter regions. Promoter region (-500/+1) of human *Sirpα* gene was aligned with the promoter region (-500/+1) of mouse *Sirpα* gene using an online software (<https://blast.ncbi.nlm.nih.gov/Blast.cgi>). The conserved sequences were marked by a red line.

**(b)** Prediction of transcriptional factor for gene *Sirpα.* The underlined conserved sequence as indicated in (**a**) were analyzed for the potential transcriptional factor using an online software (<http://alggen.lsi.upc.es/>). Multiple transcriptional factor candidates were displayed.

**(c)** Confirmation of transcriptional factors for *Sirpα.* Raw cells were transfected with a scramble shRNA (sh-con) or a shRNA specifically targeting mouse Elk-1, C/EBPb, YY1 or TFIID, respectively. 24 h later, cells were harvested for Real-time PCR assays of *Sirpα* (n=4)

**(d)** TFIID mRNA levels in TAMs. MC-38 cells (1.0 × 10^6^/100 μl PBS) were subcutaneously injected into 6-week-old WT C57BL/6 mice. One or two weeks later, the TAMs were isolated and subjected to Realtime PCR assays. (n=3)

Data in (**c,d**) showed means±s.e.m. (*P<0.05, ***P<0.005; ns, not significant Student’s *t* test)


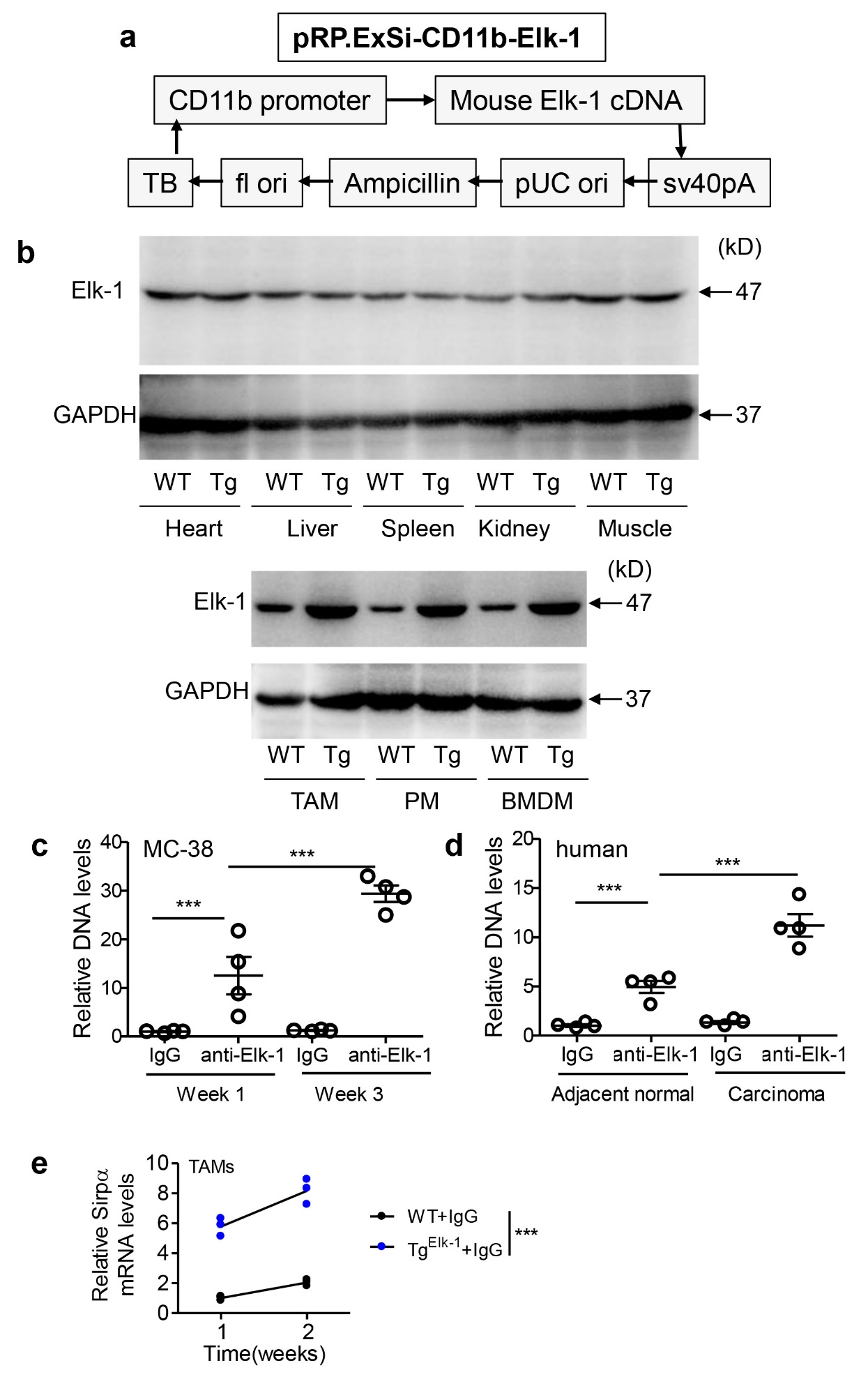


Figure. S3.

**Validation of myeloid transgene of *Elk-1* in mice**

**(a)** Schematic diagram for CD11b promoter-driven transgene of mouse Elk-1 (Tg^Elk-1^).

**(b)** Western blotting assays of Elk-1 in multiple tissues and macrophages from WT and Tg^Elk-1^ mice. TAM, tumor associated macrophage; PM, peritoneal macrophage; BMDM, bone marrow-derived macrophage. TAMs were isolated from MC-38 cell-based subcutaneous xenograft models.

**(c)** Interactions between Elk-1 protein and Sirpα promoter DNA in mouse TAMs from subcutaneous MC-38 tumors at different time points were assessed by ChIP assays.(n=4)

**(d)** Interactions between Elk-1 protein and Sirpα promoter DNA in human TAMs from adjacent normal or carcinoma tissues were assessed by ChIP assays. (n=4)

**(e)** GFP-tagged MC-38 cells (1.0 × 10^6^/100 μl of PBS) were subcutaneously inoculated into 6-week-old WT or Tg^Elk-1^ mice. Each mouse was treated (i.v.) with IgG as control on days 1, 3 and 5 at a dosage of 100 μg/day. One or two weeks later, the tumors were collected and Sirpα mRNA levels were measured by Realtime PCR assays (n=3).

Data in (**c-e**) showed means±s.e.m. (***P<0.005; Student’s *t* test)


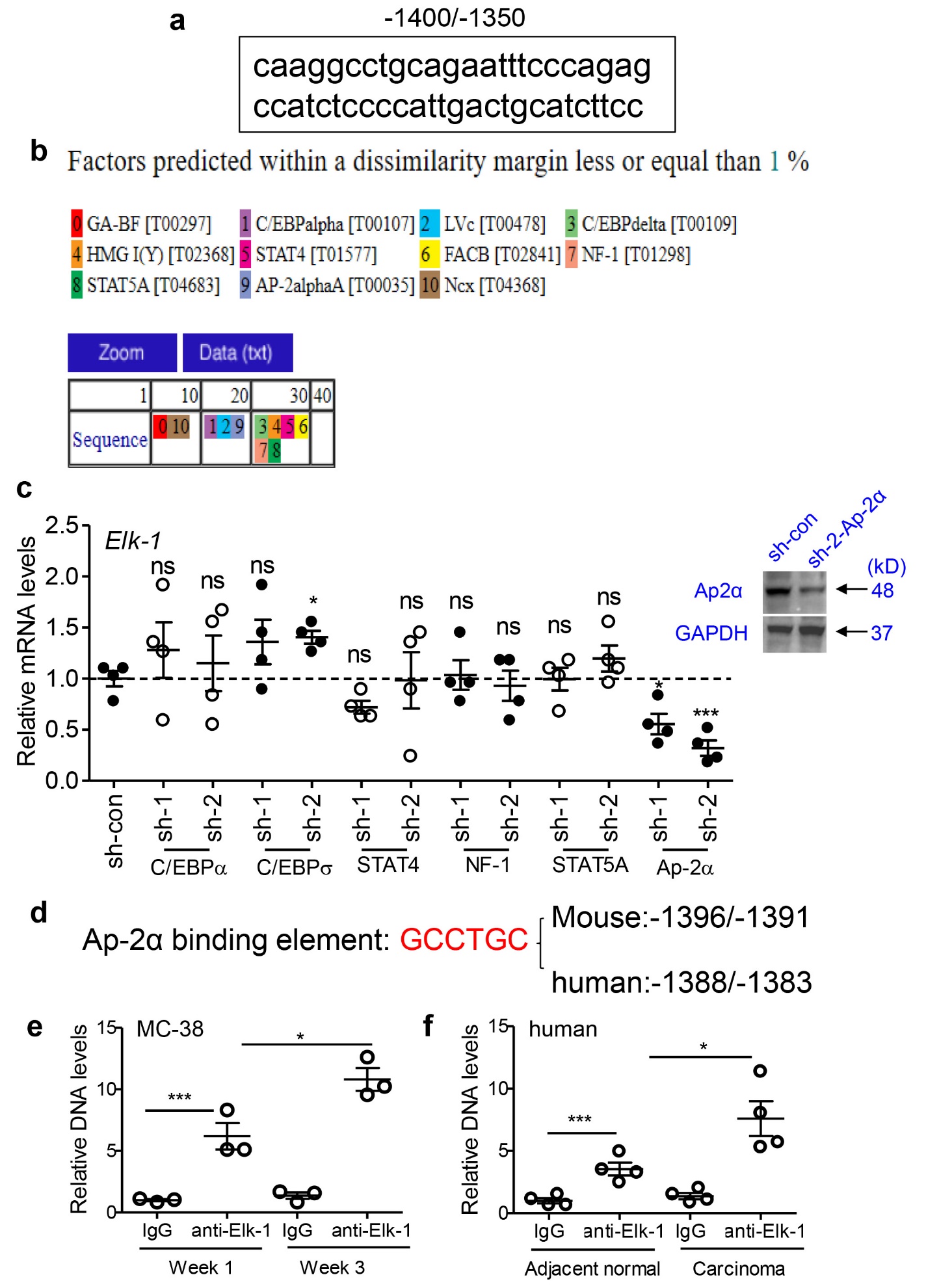


**Figure. S4.**

**Prediction of transcriptional factor for gene *Elk-1***

**(a)** The sequences of a functional promoter region locating -1450/-1300.

**(b)** Prediction of transcriptional factor for gene *Elk-1.* The sequences indicated in (**a**) were analyzed for the potential transcriptional factor using an online software (<http://alggen.lsi.upc.es/>). Multiple transcriptional factor candidates were displayed.

**(c)** Validation of transcriptional factors for *Elk-1.* Raw cells were transfected with a scramble shRNA (sh-con) or a shRNA specifically targeting mouse C/EBPα, C/EBPδ, STAT4, NF-1, STAT5A or Ap-2α, respectively. 24 h later, cells were harvested for Real-time PCR assays of *Elk-1* (n=4). The knockdown efficiency of sh-2-Ap-2α was measured by western blotting assays.

**(d)** A potential Ap-2α binding element (GCCTGC) locating -1396/-1391 in mouse *Elk-1* and -1388/-1383 in human *ELK-1*.

**(e)** Interactions between Ap-2α protein and Elk-1 promoter DNA in mouse TAMs from subcutaneous MC-38 tumors at different time point were assessed by ChIP assays. Data showed means±s.e.m. (n=3)

**(f)** Interactions between Ap-2α protein and Elk-1 promoter DNA in human TAMs from adjacent normal or carcinoma tissues were assessed by ChIP assays. (n=4)

Data in (**c**), (**e**) and (**f**) showed means±s.e.m. (*P<0.05, ***P<0.005; Student’s *t* test)


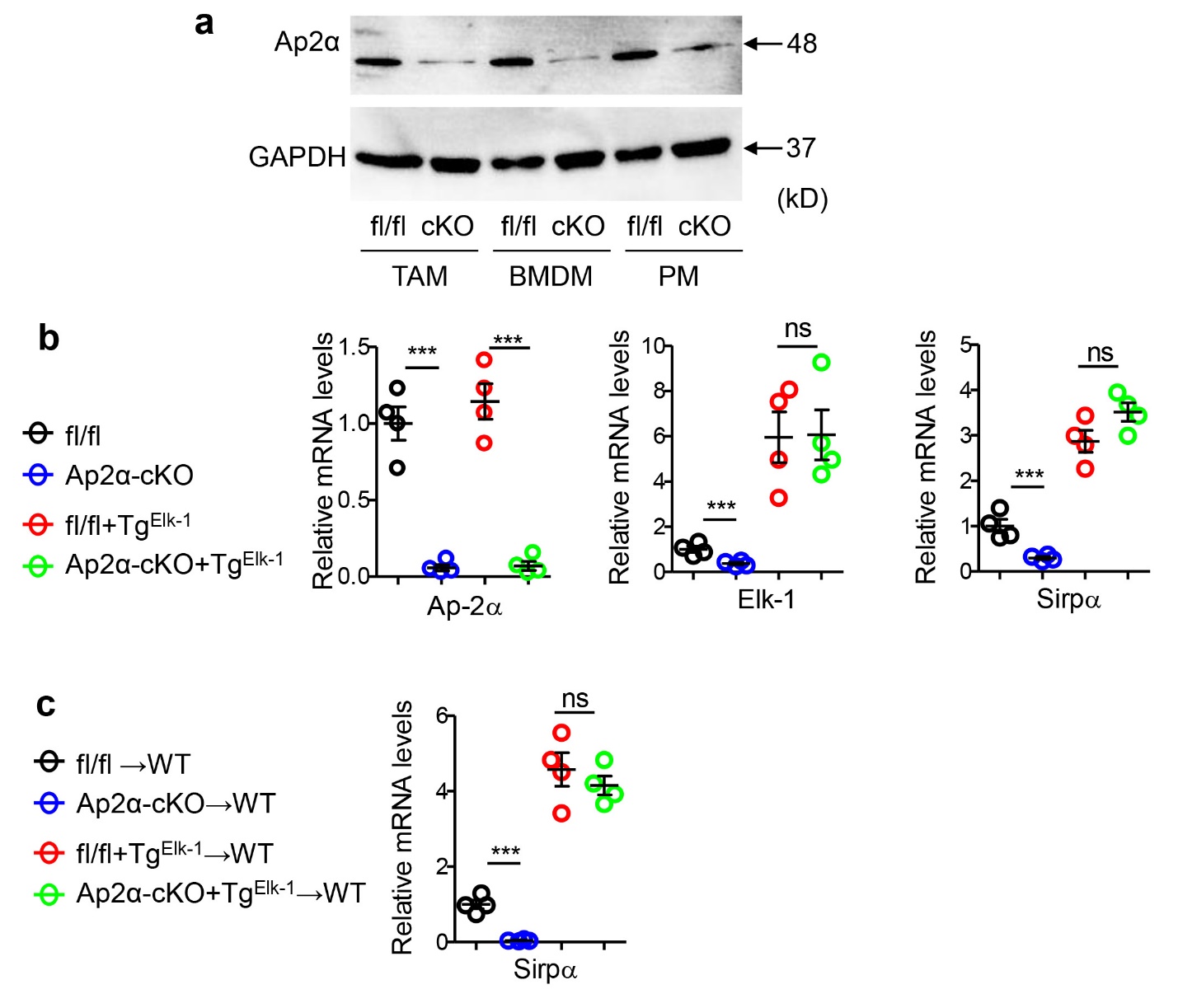


**Figure. S5.**

**Validation of Ap-2α knockout in macrophages and Sirpα expression in TAMs**

**(a)** TAMs, BMDMs and PMs were collected from Ap2α-cKO or fl/fl mice and subjected to Western blotting assays of Ap-2α.

(**b**) mRNA levels of Ap-2α, Elk-1 and Sirpα in the PMs from the fl/fl, Ap2α-cKO, fl/fl+Tg^Elk-1^ and Ap2α-cKO+Tg^Elk-1^ mice.

(**c**) Six-week-old male fl/fl, Ap2α-cKO, fl/fl+Tg^Elk-1^ and Ap2α-cKO+Tg^Elk-1^ mice were subcutaneously engrafted with GFP-tagged MC-38 cells (1.0 × 10^6^ cells per mouse). Two weeks later, the TAMs were isolated and subjected to Realtime PCR assays of Sirpα mRNA levels.

Data in (**b,c**) showed means±s.e.m. (n=4, ***P<0.005; ns, not significant; Student’s *t* test)


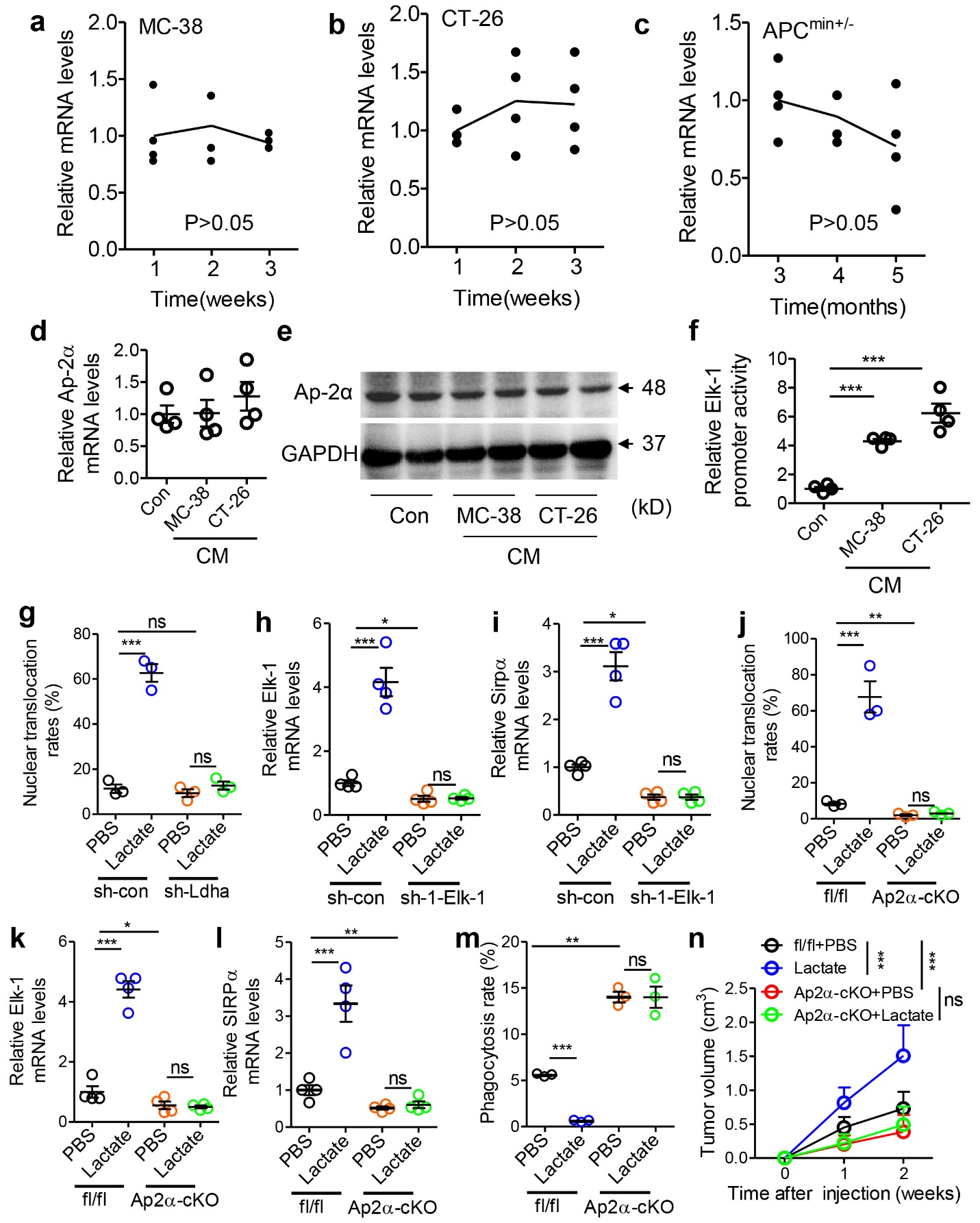


**Figure. S6.**

**Lactate regulates Ap-2α/Elk/Sirpα axis in TAMs**

**(a)** Ap-2α mRNA levels in TAMs from subcutaneous MC-38 tumor models. Six-week-old male C57BL/6 mice were subcutaneously inoculated with MC-38 cells (1.0 × 10^6^/100 μl PBS). TAMs were isolated for Real-time PCR assays of Ap-2α dynamically. (n=3)

**(b)** Ap-2α mRNA levels in TAMs from subcutaneous CT-26 xenograft models. Six-week-old male BALB/c mice were subcutaneously engrafted with CT-26 cells (1.0 × 10^6^/100 μl PBS). TAMs were isolated for Real-time PCR assays of Ap-2α dynamically. (n=3)

**(c)** Ap-2α mRNA levels in TAMs from APC^min+/-^ mice. Primary tumor nodes were dissected at the age of 3, 4 and 5 months. TAMs were isolated for Real-time PCR assays of Ap-2α. (n=5)

**(d)** Relative mRNA levels of Ap-2α in the PMs treated with MC-38- or CT-26-conditioned medium for 24 h. (n=4)

**(e)** Western blotting assays of Ap-2α in the PMs treated with MC-38- or CT-26-conditioned medium for 24 h.

**(f)** Relative Elk-1 promoter activity in the PMs treated with MC-38- or CT-26-conditioned medium for 24 h. (n=4)

**(g)** Peritoneal macrophages were transfected with an Ldha-specific shRNA (sh-Ldha) or sh-con as control for 24 h and then stimulated with lactate (10 mM) or PBS for 24 h. Then, the cells were stained with anti-Ap-2α antibodies and nuclear translocation rates were evaluated. (n=3)

(**h,i**) Peritoneal macrophages were transfected with an Elk-1-specific shRNA (sh-Elk-1) or sh-con as control for 24 h and then stimulated with lactate (10 mM) or PBS for 24 h. Then, the cells were collected for Realtime PCR assays of Elk-1(**h**) and Sirpα (**i**).(n=4)

(**j**) Peritoneal macrophages from fl/fl and Ap-2α-cKO mice were stimulated with lactate (10 mM) or PBS for 24 h. Then, the cells were stained with anti-Ap-2α antibodies and nuclear translocation rates were evaluated. (n=3)

(**k,l**) mRNA levels of Elk-1(**k**) and Sirpα (**l**) in the fl/fl and Ap-2α-cKO macrophages stimulated with lactate (10 mM) or PBS for 24 h.(n=4)

(**m**) The tumor phagocytosis rates of fl/fl and Ap-2α-cKO macrophages treated with lactate (10 mM) or PBS for 24 h were evaluated. (n=3)

(**n**) Six-week-old male fl/fl and Ap-2α-cKO mice were subcutaneously engrafted with MC-38 cells (1.0 × 10^6^ cells per mice) and then subcutaneously treated with lactate (50 μmol in 100 μl of PBS per injection) or PBS on days 1, 3 and 5. Tumor volume was measured dynamically. (n=10)

All data indicated means±s.e.m. Data in (**a-c**) were analyzed by One-way ANOVA. Data in (**d**) and (**f-n**) were analyzed by Student’s *t* test (*P<0.05, **P<0.01, ***P<0.005; ns, not significant).

**
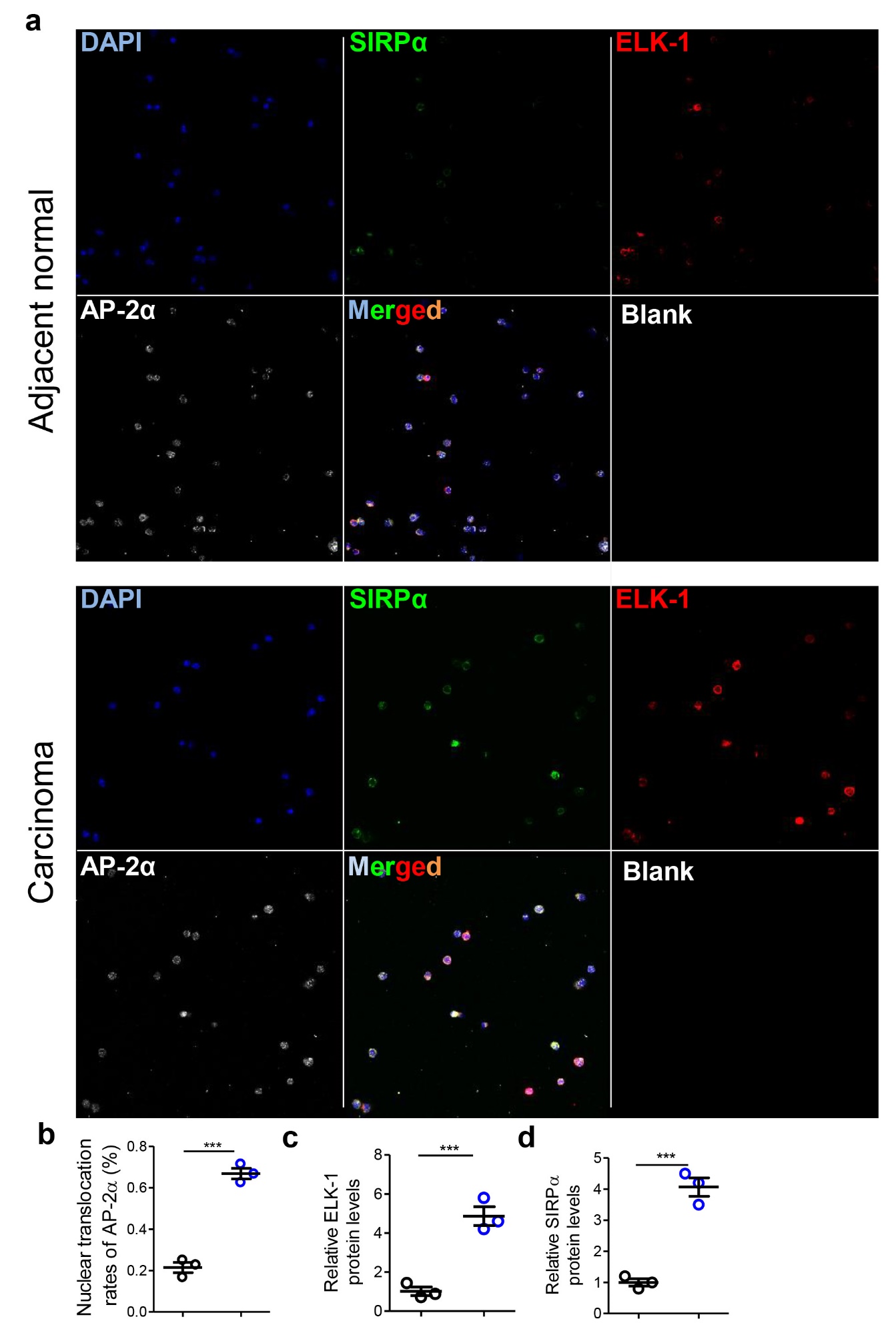
**

**Figure. S7.**

**Validation of Ap-2α/Elk/Sirpα axis in human TAMs**

(**a**) Macrophages from carcinoma or adjacent normal tissues of CRC patients were isolated and subjected to immunofluorescent staining of SIRPα (green), ELK-1(red) and AP-2α (white). The nuclei were visualized by DAPI staining (blue). Representative images were displayed.

(**b-d**) The nuclear translocation rates (**b**) as well as expression of ELK-1(**c**) and SIRPα (**d**) in the TAMs as described above in (**a**) were calculated. Data indicated means±s.e.m. and were analyzed by Student’s *t* test (n=3, ***P<0.005).

Table S1.

**shRNA sequences for gene silencing**.

| Gene | Species |  | shRNA sequences |
| --- | --- | --- | --- |
| Ap-2α | mouse | #1 | CCGGGCAGAATTTCTCAACCGACAACTCGAGTTGTCGGTTGAGAAATTCTGCTTTTT |
|  |  | #2 | CCGGCGGAGAGCGAAGTCTAAGAATCTCGAGATTCTTAGACTTCGCTCTCCGTTTTT |
| Elk-1 | mouse | #1 | CCGGGCTGTCAAAGCTGAACCAGAACTCGAGTTCTGGTTCAGCTTTGACAGCTTTTTG |
|  |  | #2 | CCGGCGGTACTACTATGATAAGAATCTCGAGATTCTTATCATAGTAGTACCGTTTTTG |
| C/EBPb | mouse | #1 | CCGGCTGACGCAACACACGTGTAACCTCGAGGTTACACGTGTGTTGCGTCAGTTTTTG |
|  |  | #2 | CCGGCACCCTGCGGAACTTGTTCAACTCGAGTTGAACAAGTTCCGCAGGGTGTTTTTG |
| YY1 | mouse | #1 | CCGGCGACGGTTGTAATAAGAAGTTCTCGAGAACTTCTTATTACAACCGTCGTTTTTG |
|  |  | #2 | CCGGCACATCTTAACACACGCTAAACTCGAGTTTAGCGTGTGTTAAGATGTGTTTTTG |
| TFIID | mouse | #1 | CCGGCCAGAATTATTTCCTGGATTACTCGAGTAATCCAGGAAATAATTCTGGTTTTTG |
|  |  | #2 | CCGGGTAGCTATGAGCCAGAATTATCTCGAGATAATTCTGGCTCATAGCTACTTTTTG |
| C/EBPα | mouse | #1 | CCGGAGCCGAGATAAAGCCAAACAACTCGAGTTGTTTGGCTTTATCTCGGCTTTTTT |
|  |  | #2 | CCGGAGCCGAGATAAAGCCAAACAACTCGAGTTGTTTGGCTTTATCTCGGCTTTTTTG |
| C/EBPδ | mouse | #1 | CCGGTCTTCAACAGCAACCACAAAGCTCGAGCTTTGTGGTTGCTGTTGAAGATTTTTG |
|  |  | #2 | CCGGTAGAGCTGTGCCACGACGAACCTCGAGGTTCGTCGTGGCACAGCTCTATTTTTG |
| STAT4 | mouse | #1 | CCGGCTTTACCATAGATCACAATTTCTCGAGAAATTGTGATCTATGGTAAAGTTTTTG |
|  |  | #2 | CCGGGCTATTGATTCACAATCTAAACTCGAGTTTAGATTGTGAATCAATAGCTTTTTG |
| NF-1 | mouse | #1 | CCGGCCCTGTAAATAGTTTGTGTAACTCGAGTTACACAAACTATTTACAGGGTTTTTG |
|  |  | #2 | CCGGGCCAACCTTAACCTCTCTAATCTCGAGATTAGAGAGGTTAAGGTTGGCTTTTTG |
| STAT5A | mouse | #1 | CCGGTTGACCAAGATGGCGAGTTTGCTCGAGCAAACTCGCCATCTTGGTCAATTTTTG |
|  |  | #2 | CCGGTGGTCCCTGAGTTCGTCAATGCTCGAGCATTGACGAACTCAGGGACCATTTTTG |
| Control |  |  | CCGGTCTAGACGCAACTACCAGAAGTGAGTCGCTAATTAGTCGAATGTTCTTTTTTTG |

Table S2.

**Basic information of patients for macrophage isolation**

| Stage | N(M/F) | Average age  (M/F,Years) | Chemotherapy before surgery(%) |
| --- | --- | --- | --- |
| T2-4N0M0 | 8/7 | 66/64 | 0 |
| T2-4N1-2M0 | 9/6 | 64/58 | 0 |
| T2-4N0-2M1 | 3/9 | 71/57 | 0 |

Table S3.

**Primer sequences for Real-time PCR**

| Gene | species | Forward (5' -> 3') | Reverse (5' -> 3') |
| --- | --- | --- | --- |
| Ap-2α | mouse | CAATGAGCAAGTGGCAAGAA | AGGGCCTCGGTGAGATAGTT |
| Elk-1 | mouse | CTGCTCCCCACACATACCTT | GAGAGGCCATCCACACTGAT |
| Sirpα | mouse | GCTTCTCTCCCCGGAATATC | CAAGGTGATGTGGGCTACCT |
| Gapdh | mouse | AACTTTGGCATTGTGGAAGG | ACACATTGGGGGTAGGAACA |
| AP-2α | human | GTTGCACCCAGGAAACCTTA | TCCTCGCAGTCCTCGTACTT |
| ELK-1 | human | CCACCTTCACCATCCAGTCT | TCTTCCGATTTCAGGTTTGG |
| SIRPα | human | CACCTGGCTCAGGCTAGTTC | ATGTGGATTCTCCAGGTTGC |
| GAPDH | human | GAGTCAACGGATTTGGTCGT | TTGATTTTGGAGGGATCTCG |

Table S4.

**Primer sequences for cloning reporter constructs**

| Constructs | species | Forward (5' -> 3') | Reverse (5' -> 3') |
| --- | --- | --- | --- |
| p-Sirpα | mouse | gggctagcgagctagggtacattttgca | ccgagatcttgcaagggtggagagtggag |
| Mut1 | mouse | actccacacacgctatttttgt | aaggacttcagtgcctcttggt |
| Mut2 | mouse | actttcccccttccccctgcat | aaggagagagtgatggtacaaa |
| pE-1 | mouse | gcgctagcccccaaaatgagtcatttat | ggcagatcttctctcagaagctgcagcag |
| pE-2 | mouse | gcgctagcaaaccaaagaagtgaaaaac | ggcagatcttctctcagaagctgcagcag |
| pE-3 | mouse | gcgctagcgcctcggttcagtatacgtt | ggcagatcttctctcagaagctgcagcag |
| pE-4 | mouse | gcgctagccaaggcctgcagaatttccc | ggcagatcttctctcagaagctgcagcag |
| pE-5 | mouse | gcgctagccagcctccaatattaaagca | ggcagatcttctctcagaagctgcagcag |
| pE-6 | mouse | gcgctagcggaagacccatcctttctgt | ggcagatcttctctcagaagctgcagcag |
| pE-7 | mouse | gcgctagcagtccccagtactgtgtaca | ggcagatcttctctcagaagctgcagcag |
| pE-8 | mouse | gcgctagcagtttttgcctagctaatta | ggcagatcttctctcagaagctgcagcag |
| pE-1-mut | mouse | actacagaatttcccagagccatct | ccttgtagttctctaagtccct |

Table S5.

**Primer sequences for ChIP assays**

| Promoter DNA | species | Forward (5' -> 3') | Reverse (5' -> 3') |
| --- | --- | --- | --- |
| Sirpα | mouse | ttaccaagaggcactgaagt | aaaagcaggaagtaaatgca |
| SIRPα | human | cgcgcctcttttccaggcgg | agtaagtgaacgcaggggga |
| Elk-1 | mouse | gcctcggttcagtatacgtt | ggaagatgcagtcaatgggga |
| ELK-1 | human | gagaactacaaggcctgcag | atgtgactcaaaggcattca |
